# Treatment of dental implant displacement into the maxillary sinus

**DOI:** 10.1186/s40902-017-0133-1

**Published:** 2017-11-25

**Authors:** Jun-Hyeong An, Sang-Hoon Park, Jeong Joon Han, Seunggon Jung, Min-Suk Kook, Hong-Ju Park, Hee-Kyun Oh

**Affiliations:** Department of Oral and Maxillofacial Surgery, School of Dentistry, Dental Science Research Institute, Chonnam National University, 77, Yongbong-ro, Buk-gu, Gwangju, 500-757 Republic of Korea

**Keywords:** Dental implant displacement, Maxillary sinus, Simultaneous treatment

## Abstract

**Background:**

Displacement of dental implants into the maxillary sinus is rare, but it primarily occurs in patients with severe pneumatization of the maxillary sinus and/or deficiency of the alveolar process. Some complications such as the infection of the paranasal sinuses and formation of the oroantral fistula can be followed by the displacement of a dental implant. Therefore, the displaced implant has to be removed immediately with surgical intervention show and another plan for rehabilitation should be considered.

**Main body:**

The conventional procedure for the removal of a displaced implant from the maxillary sinus involves sinus bone grafting and new implant placement performed in two or more steps with a significant time gap in between. Simplification of these surgical procedures can decrease the treatment duration and patient discomfort.

**Conclusions:**

In this review, we discuss the anatomical characteristics of the maxillary sinus and the complications associated with implant displacement into the sinus.

## Introduction

Severe maxillary sinus pneumatization and thin residual alveolar bone can lead to the displacement of dental implants into the maxillary sinus during placement or after prosthetic restoration. Biting forces on the implant prosthesis and surrounding structures can also result in displacement of the implant. Displacement can occur more often in patients who undergo simultaneous implant placement and bone grafting after sinus elevation [1]. Another factor is the decreased height of the residual alveolar bone, in particular implant placement in bone with a minimal height of less than 4 mm with simultaneous bone grafting through sinus elevation [2]. Galindo et al. stated that differences in the air pressure between the maxillary sinus and nasal cavity and an autoimmune reaction to dental implants, which causes bone resorption due to peri-implantitis, can also result in implant displacement [3]. Other influencing factors include the lack of the primary stability, the surgeon’s lack of experience, temporary denture usage without relief, overdrilling, and the inappropriate application of force during the removal of nonintegrated implants [4].

Immediate removal of displaced implants is usually recommended. However, when the removal is delayed, the sinus infection needs to be controlled by antibiotics and nasal decongestants before removal of the implant using Caldwell-Luc approach or an endoscopic technique [5]. After removal of the displaced implant, implant placement with bone grafting can be performed stage by stage. However, this conventional approach is extremely long and delays appropriate rehabilitation of the edentulous area.

In this review, we discuss the anatomical characteristics of the maxillary sinus, conventional approach for the removal of displaced implants from the maxillary sinus, and complications associated with implant displacement and describe a simplified treatment process for the removal of displaced implants with simultaneous sinus bone grafting and new implant placement.

## Review

### Anatomical characteristics of the maxillary sinus

The maxillary sinus is a pyramid-shaped cavity lined with mucoperiosteum-containing cilia. The base is at the lateral nasal wall and the apex is toward the zygomatic bone and zygomatic arch. The maxillary sinus is connected to the nasal cavity through the maxillary ostium, which is an opening into the nasal cavity. This opening is relatively far from the floor of the maxillary sinus. The mucoperiosteal lining of the maxillary sinus is known as the Schneiderian membrane, which is approximately 1.0-mm thick. The posterior superior alveolar nerve and vessels usually pass through the maxillary sinus, and the average distance from the nerve to the crestal ridge of the alveolar bone is 16.9 mm [6–8].

The maxillary sinus in adults measures 25–35 mm in width, 36–45 mm in height, and 38–45 mm in length [8]. As time passes after the loss of maxillary teeth, the maxillary sinus generally expands to fill in the space through resorption of the alveolar bone. This so-called pneumatization caused by basal bone loss due to reinforced osteoclastic activity in the maxillary sinus membrane [9, 10] can lead to perforation of the maxillary sinus membrane. The other most common factor that contributes to perforation of the sinus membrane is the presence of septa, which exhibits an incidence rate of 31.7% for the premolar region [11].

### Complications associated with dental implant displacement into the maxillary sinus

Dental implant displacement into the maxillary sinus may be an intraoperative or postoperative complication. The displaced implant can disturb the anatomy around the maxillary sinus and inhibit mucociliary clearance by the cilia in the sinus membrane [12]. Furthermore, mucosal thickening may occur, and scattered bone graft material may obstruct maxillary ostium to result in maxillary sinusitis and congestion [13].

In the event of maxillary sinusitis and blockage of the ostium, an oroantral fistula can develop [11]. Alberto et al. described that accidentally displaced implants can also migrate from the maxillary sinus to the upper structures such as the paranasal sinuses, orbital floor, or cranial fossa through mucociliary clearance against the force of gravity, changes in the air pressure of the nasal cavity, a foreign body reaction, and local tissue necrosis [14].

Some studies have described that implants displaced into the maxillary sinus may not result in maxillary sinusitis [15, 16]. Galindo-Moreno et al. reported two cases of antral implant migration. The migrated implant that had been left behind on request of the patient showed no signs of clinical complications at 4-year follow-up visit [17]. On the other hand, Regev et al. and Raghoebar et al. suggested that the displaced dental implants in the maxillary sinus result in chronic maxillary sinusitis because of a foreign body reaction and need to be eliminated through surgical intervention, even if the patient is asymptomatic [1, 12, 18].

### Treatment modalities for a compromised maxillary sinus containing displaced dental implants

As aforementioned, the basic principle is immediate removal of the displaced implant. However, when removal is delayed, the sinus infection should be controlled at the patient’s first visit. To prevent mucosal thickening and maxillary sinusitis, amoxicillin with clavulanate and nonsteroidal anti-inflammatory drugs need to be prescribed with pseudopehpedrine hydrochloride for 1 week before surgery [19].

Initially, management strategies for displaced dental implants in the maxillary sinus included the Caldwell-Luc procedure and conservative observation in the absence of signs or symptoms [12]. Lately, minimally invasive maxillofacial surgery is preferred, with functional endoscopic sinus surgery (FESS) and conservative intraoral surgery with the formation of a bony window in the lateral wall of the maxillary sinus being representative procedures [20–22]. Tsodoulos et al. reported a case involving a patient who was operated under general anesthesia using a minimally invasive approach. A small rectangular bony window in the lateral wall of the maxillary sinus was created under direct vision. After the removal of the displaced implant from the maxillary sinus, the removed bony window was repositioned at the end of the surgery as it was guided by holes and stabilized by absorbable sutures [23]. Nogami et al. reported a case involving a patient who was operated under local anesthesia. Four holes were created and osteotomy was performed using piezoelectric instruments. Following removal of the bony fragment, the displaced implant was identified with rigid endoscope and removed by dental suction. The bony fragment was repositioned and fixed with absorbable sutures [24]. Subsequently, shorter and wider implants (by 1–2 mm) can be placed with or without sinus bone grafting after confirmation of the residual alveolar bone height and width on cone beam computed tomography images obtained 4–6 months later [25–30].

In the most of the reported studies mentioned above, removal of the displaced implant, sinus bone grafting, and new implant placement were divided into two or three individual procedures. Delayed implant placement is usually indicated when primary stability cannot be obtained or when extensive perforation of the sinus membrane or severe sinus infection is present [2]. However, these divided procedures delay rehabilitation of the edentulous area.

### Surgical strategy for removal and sinus bone graft with simultaneous implant placement

**Fig. 1:**
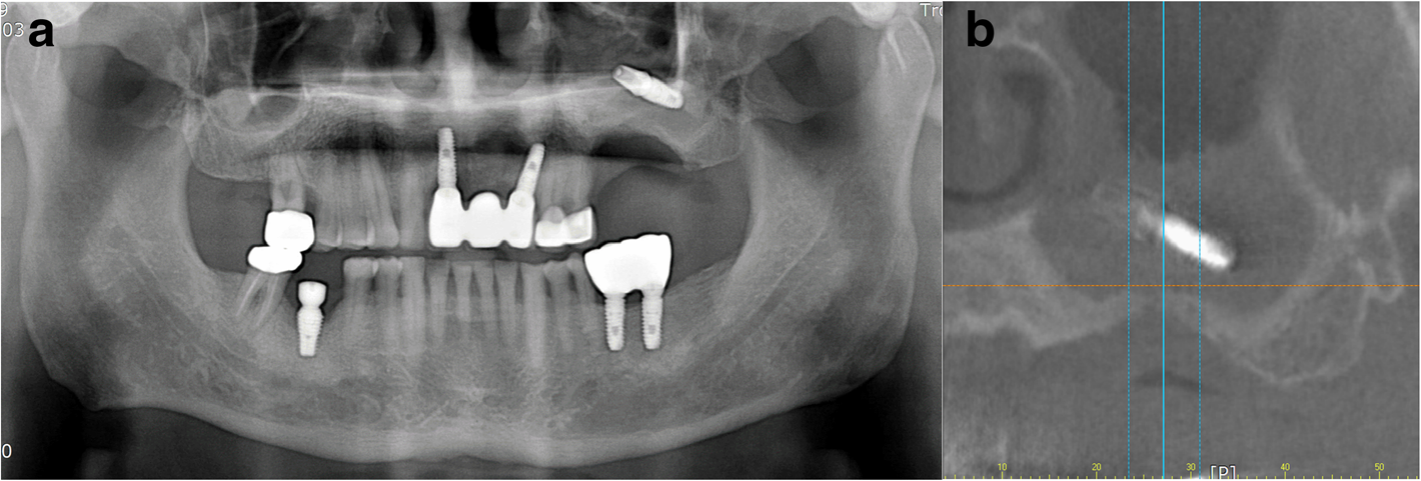
Preoperative **a** panoramic view and **b** CBCT view

**Fig. 2:**
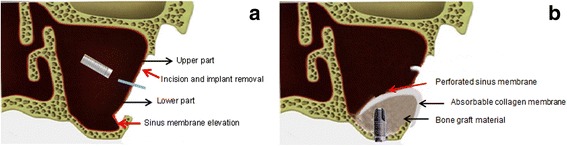
Illustration describing the bony window extended upward. **a** The upper part is used for removal of displaced implant and the lower part is used for elevation of sinus membrane. **b** The perforated sinus membrane was covered with absorbable collagen membrane and implants placed with sinus bone graft

**Fig. 3:**
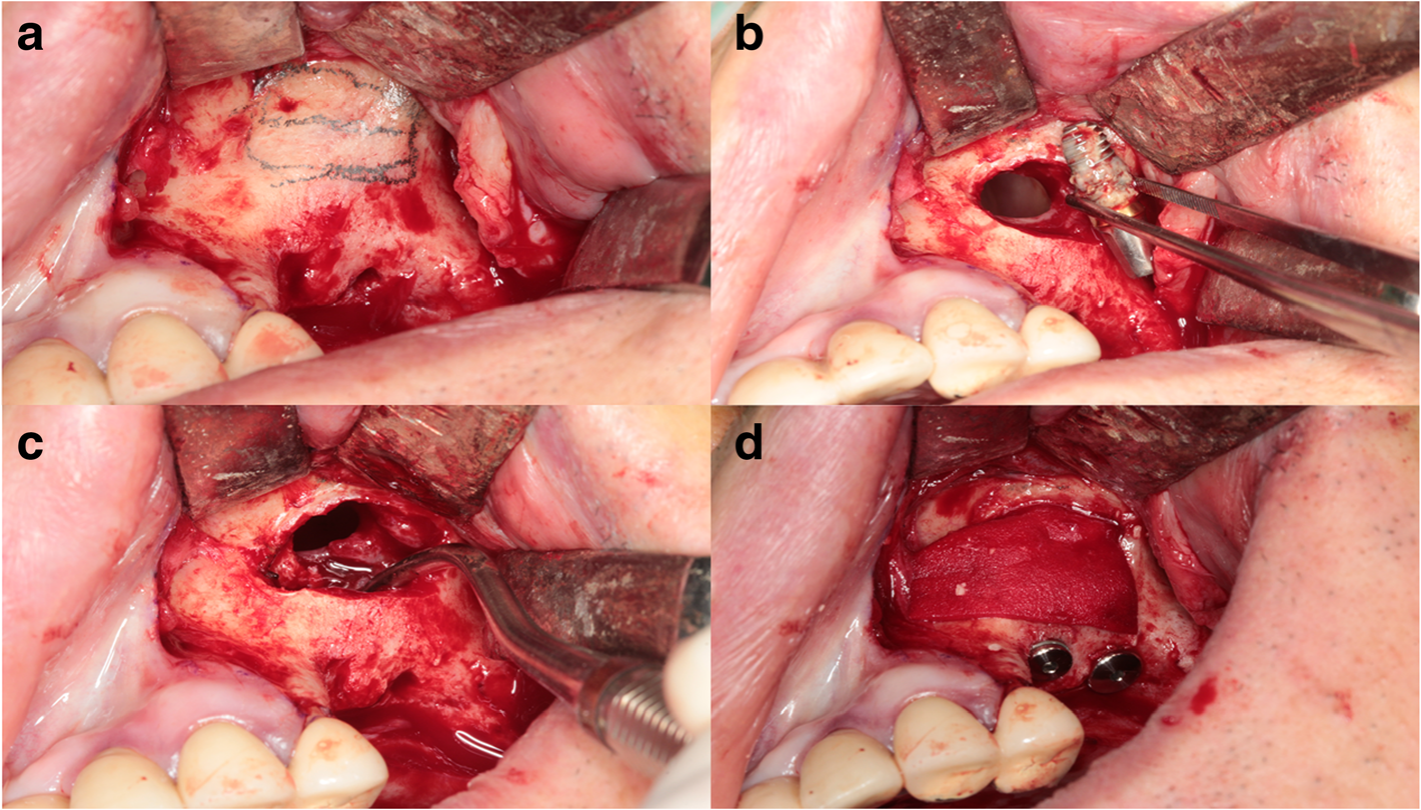
Intraoperative photographs. **a** Marking of bony window. **b** Removal of the displaced implant through the upper part. **c** Sinus elevation through the lower part. **d** Implant installation with sinus bone graft using absorbable membrane and fibrin glue

A 63-year-old man who complained of pain on the left cheek area was referred from local dental clinic for removal of a displaced dental implant which was placed 3 years ago. Panoramic radiograph, cone beam computed tomography (CBCT) scans disclosed a dental implant in the left maxillary sinus with mucosal thickening maxillary sinusitis (Fig. 1). The operation was done under local anesthesia. The surgical intervention began with elevation of full-thickness mucoperiosteal flap. After exposure of lateral wall of the maxillary sinus, the bony window was marked by ditching with a round bur. The size of bony window was vertically wider than usual bony window in maxillary sinus elevation. The upper portion is for removal of the displaced implant, while the lower portion is for lifting of the maxillary sinus membrane (Fig. 2). Following exposure of the sinus membrane and removal of the bony window, a horizontal incision is placed at the upper portion of the bony window. Through this opening, the implant is removed using dental suction. Then, the sinus membrane is lifted, starting from the lower edge of the bony window. The perforated sinus membrane is covered with absorbable collagen membrane, and the new implant is placed simultaneously with sinus bone grafting using a mixture of graft from the maxillary tuberosity and allograft (Fig. 3).

**Fig. 4:**
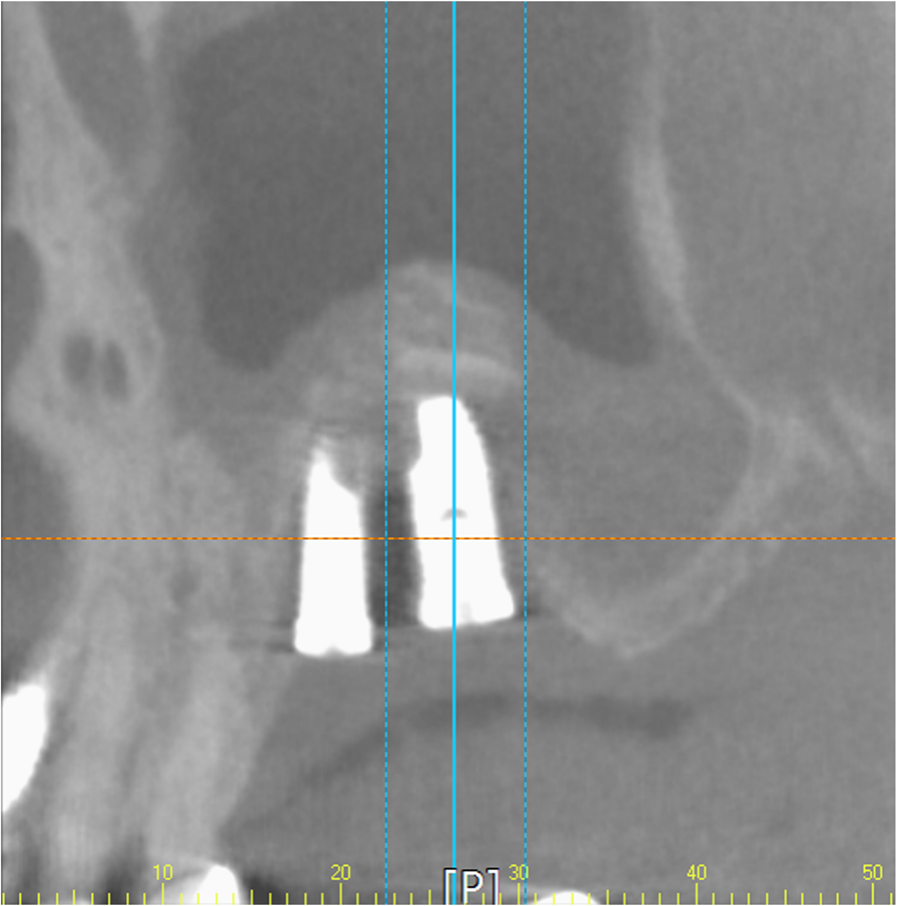
Postoperative CBCT view 4 months later

**Fig. 5:**
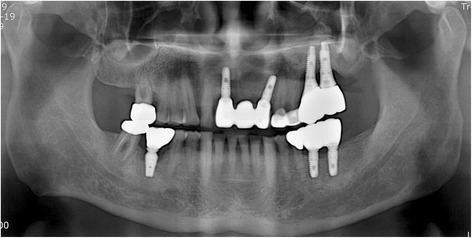
Postoperative panoramic view 14 months later

After the surgery, patients are instructed to avoid blowing their nose for 2 weeks and sneeze with the mouth open. The routine postoperative medications are prescribed for 1 week to prevent sinus infection [31]. No haziness was observed in the CBCT taken 4 months later (Fig. 4). The secondary surgery was done 6 months later after implant placement. Prosthetic restoration of the implants was done in 2 months later. Until now, there have been no complications during postoperative 14 month follow-up period (Fig. 5).

## Conclusions

Dental implants displaced into the maxillary sinus should be removed immediately. However, immediate removal is occasionally not possible because of the patient’s condition or the dentist’s lack of technical experience. In such cases, maxillary sinusitis should be controlled with proper measures, including antibiotics and nasal decongestants, before surgical intervention. Subsequently, implant removal and simultaneous new implant placement with sinus bone grafting can be performed through an extended bony window. This new approach decreases effort, time, and patient discomfort and accelerates the rehabilitation process.
